# Nuclear archaeology reassesses Heisenberg’s last reactor experiment

**DOI:** 10.1093/pnasnexus/pgag282

**Published:** 2026-09-29

**Authors:** Patrick J Park, Brittany Robertson, Miriam E Hiebert, Jason Zhao, Ciara B Sivels, Timothy W Koeth

**Affiliations:** Department of Applied Physics, Columbia University, New York, NY, USA; Department of Physics, Reed College, Portland, OR, USA; Pacific Northwest National Laboratory, Richland, WA, USA; Department of Materials Science and Engineering, University of Maryland, College Park, MD, USA; Department of Computer Science, Texas A&M University, College Station, TX, USA; Johns Hopkins University Applied Physics Laboratory, Laurel, MD, USA; Department of Materials Science and Engineering, University of Maryland, College Park, MD, USA

**Keywords:** document-based nuclear archaeology, graphite, heavy water, World War II, Heisenberg

## Abstract

The B8 pile, built in April 1945, was a natural uranium, heavy water reactor experiment and the culmination of Nazi Germany’s nuclear program. Its designer, Werner Heisenberg, claimed in a 1947 *Nature* article that the B8 was “just insufficient” in achieving self-sustaining fission, lacking only a “relatively small amount of uranium.” In the following decades, historians have then suggested that the Germans should have pursued graphite reactors like the Americans. Here, we present forensics and document-based archaeology of the B8’s uranium, heavy water, and graphites. We create a neutronics model that is validated by in situ 1945 measurements. Our calculations show that the B8, as built, had $k_{eff}=0.94$. To achieve self-sustaining fission at $k_{eff}=1.0$, the Germans would have needed about 2.0 times the uranium and about 2.2 times the heavy water than they used, far exceeding Heisenberg’s predictions; these inventories were about 1.2 and 1.8 times, respectively, Germany’s estimated available wartime inventories. Furthermore, we determine that their graphite, with 3.4 wppm boron impurities and re-used from the prior B7 experiment, would not allow criticality in a hypothetical graphite-natural uranium pile. German graphites were made of metallurgical coke, which naturally carry more boron than petroleum coke-based graphites, which were available in America and uniquely enabled Chicago Pile 1’s criticality. Our findings indicate that Germany could not have built either a heavy water nor a graphite reactor in World War II.

Significance statement The B8 was Nazi Germany’s final nuclear reactor experiment in 1945. It fell short of criticality, i.e. a self-sustaining fission chain reaction to power a working reactor, which its designer Werner Heisenberg claimed was due to lacking only marginally more uranium. Our new nuclear analysis reveals that the B8 required roughly 2 times the uranium and 2.3 times the heavy water to reach criticality, far exceeding Heisenberg’s estimates. Additionally, graphite—as an alternative to heavy water—was unusable for reactor purposes as they were produced from coal in Germany, which naturally contain far more neutron-absorbing boron impurities than petroleum-based graphites available in America. We conclude Germany could not have built heavy water nor graphite reactors during World War II.

## Introduction

The story of our nuclear age usually starts with the famous letter from Leo Szilard and Albert Einstein to President Roosevelt on August 2, 1939. Yet, this letter quite explicitly urges starting the *second* atomic project—the first being Germany’s, already underway since April 1939. While the Manhattan Project casts a long shadow over nuclear history today, from 1939 to 1942, the Americans primarily framed their own program as needing to “catch up” to a perceived German lead (1). Ironically, despite these fears motivating the Americans for half the war, there are almost no technical evaluations of the German Uranprojekt today.

This gap stems partly from the Germans’ deprioritization of nuclear research after military setbacks in 1942, and partly because much of their original data was classified as part of the findings of the US Alsos Mission until the 1970s. In the chaos following Germany’s surrender, their nuclear materiel also disappeared into American or Soviet intelligence collections or onto the black market (2). This left a window for surviving scientists like Werner Heisenberg to make certain technical claims about their project which could not have been independently verified. For instance, Heisenberg wrote in *Nature* after the war that the material available at Haigerloch for his final reactor experiment—the B8 pile in April 1945—“was just insufficient” to attain criticality, and that “a relatively small amount of uranium would in all probability have sufficed” (3).

His claims divide modern consensus on why Germany failed to build a critical reactor. Some take Heisenberg’s claims at face value, believing that the German choice of heavy water moderation would have succeeded had it not been for slight shortages of it. Others, like Richard Rhodes, of *Making of the Atomic Bomb* fame, argue that Germany fundamentally erred by not pursuing graphite-moderated reactors (4). It is worth noting that for many other wartime German programs—such as the V-2 rockets or jet engines—21st-century scholars have critically revised embellishments found in earlier narratives (5, 6). But, a comparable re-evaluation of the Uranprojekt has lagged, leaving many historical assessments contingent on unverified postwar claims.

In this article, we present new document-based archaeological findings that narrow down the quantity and purity of the Uranprojekt's graphite and heavy water, plus forensic analyses of their uranium density. These enable construction of a neutronics model of the B8 pile that accurately reproduces 1945 German measurements while significantly reducing the modeling assumptions in prior works. Using this validated model, we calculate minimum critical reactor configurations to quantitatively assess Heisenberg’s claims. We further evaluate hypothetical graphite piles and show that neither the heavy water B8 nor the graphite piles could have achieved criticality with the materials present in wartime Germany. Our benchmarks offer improved calibration data to critically revise both scientific and historical understandings of early nuclear programs.

## Heisenberg and pile experiments

In April 1939—just months after the discovery of nuclear fission in Berlin—the Reich Research Council and the Army Ordnance Office independently convened groups of physicists to explore the implications of fission. When war broke out on 1939 September 1, the military formally took control of all nuclear research—nearly 3 years before the equivalent Manhattan District was formed (7). Leading scientists were invited to this *Uranverein* (“uranium club”), which launched experiments with graphite, dry ice, water, and heavy water. Werner Heisenberg led pile projects both at his home institution—the University of Leipzig—as well as a second with Karl Wirtz at the Kaiser Wilhelm Institute for Physics (KWIP) in Berlin. A third group worked at the Army’s proving grounds in Gottow, 50 km south of Berlin. These piles were designated by prefix “L,” “B,” or “G,” respectively, e.g. L4, B8, and G3. After Allied bombing destroyed Berlin in 1944, Heisenberg and other KWIP physicists evacuated to the southwestern village of Haigerloch, where they built and tested the B8 in April 1945.

The B8 had a right cylindrical core of radius 61.7 cm and height 124 cm with 664 cubes suspended as alternating chains of 8 and 9 cubes in 5 concentric rings (8). The remaining volume in the core, subtracting the cubes, left 1,400 L for the heavy water moderator.^a^ This inner volume was held in a 3 mm-thick shell of “Elektron” alloy, similar in composition to AZ91 Mg alloy today (9). Around the Mg was about 40 cm of graphite in each direction, then a 4 mm-thick shell of aluminum alloy, and finally 60 cm of light water on all sides. A Ra-Be or Rn-Be fast neutron source lay in the center.

To quantify neutron multiplication, Heisenberg and Wirtz measured a *Vermehrungsfaktor* (“multiplication factor”) *Z*, defined as the ratio of the thermal neutron population in the graphite plus light water reflector regions when the core loaded with uranium and heavy water ($N_{a}$) vs. that with the core empty ($N_{0}$). At the critical size, the source neutrons would multiply without limit and Z→∞. In the B8, they reported:


(1) $$Z=\frac{N_{a}}{N_{0}}=6.7.$$

From *Z*, a “neutron production coefficient” $\bar{\nu }$ was derived by neutron balance: in an empty core, the Ra-Be or Rn-Be neutron source alone replenishes neutrons absorbed in the light water reflector at a rate $N_{0}\nu_{H2O}$, where $\nu_{H2O}=3,920\;s^{-1}$ is the thermal neutron absorption rate coefficient of light water. With the core loaded, the excess population $\left(N_{a}-N_{0}\right)$ in the light water must have been produced by uranium fission, giving $\bar{\nu }=\nu_{H2O}(N_{a}-N_{0})/N_{i}$, where $N_{i}$ is the thermal neutron population in the core interior (10, 11). In the B8, the core’s neutron production rate coefficient $\bar{\nu }=430\;s^{-1}$; a positive value confirms the arrangement is neutron-producing.^b^ After the war, Wirtz derived $\bar{\nu }$’s relationship to the familiar, modern infinite multiplication factor $k_{\infty }$ by:


(2) $$\bar{\nu }=\frac{k_{\infty }-1}{\ell }\;[{\text{s}}^{-1}],$$

where ℓ is the mean neutron lifetime in the reactor (12). Using $\bar{\nu }=430\;s^{-1}$ and $\ell \approx 2.6\times 10^{-4}$ s, Wirtz translated his criticality measurement in the B8 to $k_{\infty }=1.11$.

$N_{a}$ , $N_{0}$, and $N_{i}$ were measured by the following procedure (13). Glass tubes filled with ${\text{Dy}}_{2}{\text{O}}_{3}$ solution^c^ as neutron activation probes were inserted vertically down to the core midplane, directly into the light water or through thin, watertight Al guide channels in the graphite and core.^d^ Heisenberg wrote that each probe, at distance *R* from the cylinder’s axis, yielded a neutron intensity already integrated along the reactor’s half-height, requiring only a radial integration over 2πR to approximate the thermal neutron population in that radial shell. ${\text{Dy}}_{2}{\text{O}}_{3}$ measurements in graphite were renormalized to light water-equivalent measurements by the ratio of absorption coefficients $\nu_{C}/\nu_{H2O}$. The graphite plus light water measurements gives $N_{a}$ or $N_{0}$, with the core filled or empty, respectively.

In a 1947 *Nature* article, Heisenberg wrote: “A [different group] at Stadtilm was allotted…a great part of the available uranium. The material available at Haigerloch, however, was just insufficient to attain [criticality]. A relatively small amount of uranium would in all probability have sufficed” (3). A year later, in the *FIAT Reviews of German Science*, he added that criticality “corresponds to an increase of the [core] volume by not quite half. In practice…since more ${\text{D}}_{2}\text{O}$ was not available, uranium would also be brought into the graphite reflector, which would probably have been sufficient to reach criticality” (10).

The wording here is interesting, as it implies that the B8 might have gone critical if the uranium diverted to Stadtilm had been available at Haigerloch. We will test the state of the B8 as it was built and then see if additional amounts of ${\text{D}}_{2}\text{O}$ or uranium would have truly sufficed.

## Nuclear archaeology

German primary sources omit most chemical specifications, so we reconstruct the remainder of the B8’s properties through nuclear forensics and document-based archaeology. Document-based nuclear archaeology is a systematic analysis of historical records, technical reports, correspondence, and other archival materials to reconstruct the specifications and provenance of nuclear materials when direct physical evidence is incomplete or unavailable (14, 15). This approach has been applied in prior studies of postwar nuclear facilities and fissile-material accounting (16, 17).

### Uranium

The cubes are natural uranium metal cut, cast, or pressed to length 5 cm (18). They are visually irregular; their edges are jagged and small notches were cut to fit the aluminum wire that tied each chain together. Almost all uranium oxide and metal used by the Uranverein were produced by the Degussa company. Of approximately 1,100 5-cm cubes possessed within the Uranverein, the location of only 14 are known today, split between personal, university, and museum collections (2, 19). We conducted forensic examinations of two extant cubes. A helium pycnometer measurement of one cube indicated a density $18.5349\pm 0.1412\;g/{\mathrm{cm}}^{3}$, lower than the nominal metallic density of $19.01\;g/{\mathrm{cm}}^{3}$, due to some internal porosity. Minimal oxidation was present on our sample since it was cut a few months before measurement, and any recent oxidation was removed before measuring. Gamma spectroscopy of another cube using a Canberra Falcon 5000 HPGe system indicated a uranium-235 mass fraction of 0.7242±0.0274%, confirming natural enrichment. Prior chemical analyses of other B8 cubes have estimated 7.1 weight parts per million (wppm) equivalent boron content (EBC) impurity in Degussa uranium (20).

### Heavy water

No original record of the B8 heavy water’s purity seems to have survived, but two primary sources bracket a likely range: In March 1944, Heisenberg’s collaborator Karl Wirtz lamented how pooling their heavy water with Diebner’s, to make 1.5 tons, had accidentally diluted their purity to 98.85% (21, 22). After the war, the Alsos Mission retrieved samples of the B8’s heavy water, in which a US National Bureau of Standards chemist E. R. Smith measured to be 96.8 mol% pure in 1947.^e^ Because we cannot ascertain when the adulteration from 98.85% to 96.8% occurred, we modeled the conservative 96.8% value and quantified the B8’s sensitivity to this purity separately. The Atomkeller Museum, built on the exact site of the B8, informed us that the ambient temperature in April in the experiment site was 10^∘^C, so all densities and cross sections were evaluated at that temperature.

To constrain the largest pile the Germans could have built, we also account for their total heavy water inventory. The only source of heavy water in Europe was from the Norsk Hydro plant in Vemork, Norway, which the Germans oversaw from April 1940 until its destruction by Allied bombing in November 1944. The chief engineer Alf Larsen reported a total of 2,840 kg of 100%-purity-equivalent heavy water were produced during the course of the war (23, 24). Of this, 185 kg was stolen by the French in 1940, 140 kg was lost when Heisenberg’s L4 experiment in 1942 exploded, and 679 kg were sunk by Norwegian commandos in 1944 (25). This implies at most 1,836 kg (1,654 L) were available in Germany by 1945, of which Heisenberg was able to take about 1,500 kg (about 1,364 L) of 100%-purity-equivalent heavy water to Haigerloch. When diluted to 96.8 mol% purity, this equals 1,545 kg (1,410 L) of water.

### Graphite

The reflector was built with 5×10×50 cm graphite blocks each of density $1.7\;g/{\mathrm{cm}}^{3}$ (10). Because rectangular blocks do not fit flushly in a cylindrical shell, gaps were filled with graphite powder for an average bulk density of $1.58\;g/{\mathrm{cm}}^{3}$. Germany did not synthesize reactor-grade graphite, necessitating us to identify the graphite brand to carefully model any natural impurities like boron, which is a deleterious neutron absorber. The graphite—like most of the B8’s structural materials—was almost certainly reused from the preceding B7 experiment and produced by Siemens-Plania (13). That late-war Siemens graphite from B7, when ashed, was measured to contain about half the impurities than an early-war batch from Siemens tested by Bothe and Jensen in 1941 (13). Bothe’s 1941 graphite was benchmarked to have about 6.6 wppm EBC in our prior work (26).

One uncertainty is that incinerating graphene can oxidize interstitial boron atoms and release them as gas, so impurity levels measured from the residual ash underestimate the boron originally present. Because ashing B7 graphite thus does not reveal its boron content, we presume it decreased proportionally with other mineral impurities, giving the B7 graphite an estimated 3.4 wppm EBC. Using a simple MCNP6.2 calculation, this EBC corresponds to a 5.5 mb cross section for capture over a Maxwellian-averaged thermal energy spectrum in carbon. These specifications nearly identically match that of commercial AGS graphite that Fermi and Szilard tested in 1940 and ruled out for nuclear applications (26).

It is worth noting certain differences in American vs. German graphite production studied in our prior work (26). In short, Americans used petroleum coke to make graphite and exported this coke to Germany until January 1940, when the British began a naval blockade (27, 28). Without this coke, Germans resorted to metallurgical cokes from the Ruhr, which had up to roughly twice the impurities.^f^ Even American coke was initially unsuitable for reactors, as interstitial boron in graphene lattices were unknown and could not be removed before 1947. So, Fermi and Szilard had also ruled out graphite moderation in February 1941 until a uniquely low-boron “Kendall coke” from Pennsylvania was found later that year, with which AGOT nuclear graphite was made (29, 30).

Meanwhile, Siemens in Germany attempted rudimentary thermal purification methods in their graphite, but any improvements were countered by degrading coal qualities as the war progressed. Postwar surveys by the US Field Information Agency, Technical (FIAT) recorded that of the four major graphite producers, Siemens Plania in Meitingen produced over half the total graphite in Germany and at the highest purity (27). Walther Bothe also called Siemens graphite, “the purest available in industrial quantities” (31). It is thus possible the B7/B8’s graphite achieved lower impurities than Bothe’s 1941 graphite if the late-war thermal purification methods were used with a particularly favorable batch of feedcoke.

## Simulation models

Using these specifications, we constructed three different simulation models of the B8 in Monte Carlo N-Particle (MCNP) code. First, the *fiducial model* is the B8 pile faithfully modeled as-built in April 1945, as shown in Fig. 1(b). Next, the *unit cell model* is a Wigner-Seitz cell of a single fuel cube and its surrounding heavy water with periodic boundary conditions to simulate an infinitely large reactor, in Fig. 1(c). An infinite reactor allows intrinsic nuclear properties of the fuel-moderator mix to be measured without confounding effects from neutron leakage. Finally, the *lattice model* is the fiducial model but with the fuel arranged in a hypothetical hexagonal close-packed (HCP) lattice, in Fig. 1(d). As detailed in the Methods, using a HCP lattice allows us to algorithmically scale the B8’s fuel and moderator inventories, bypassing the irregular cube-chain arrangements in the fiducial core.

**Figure 1:**
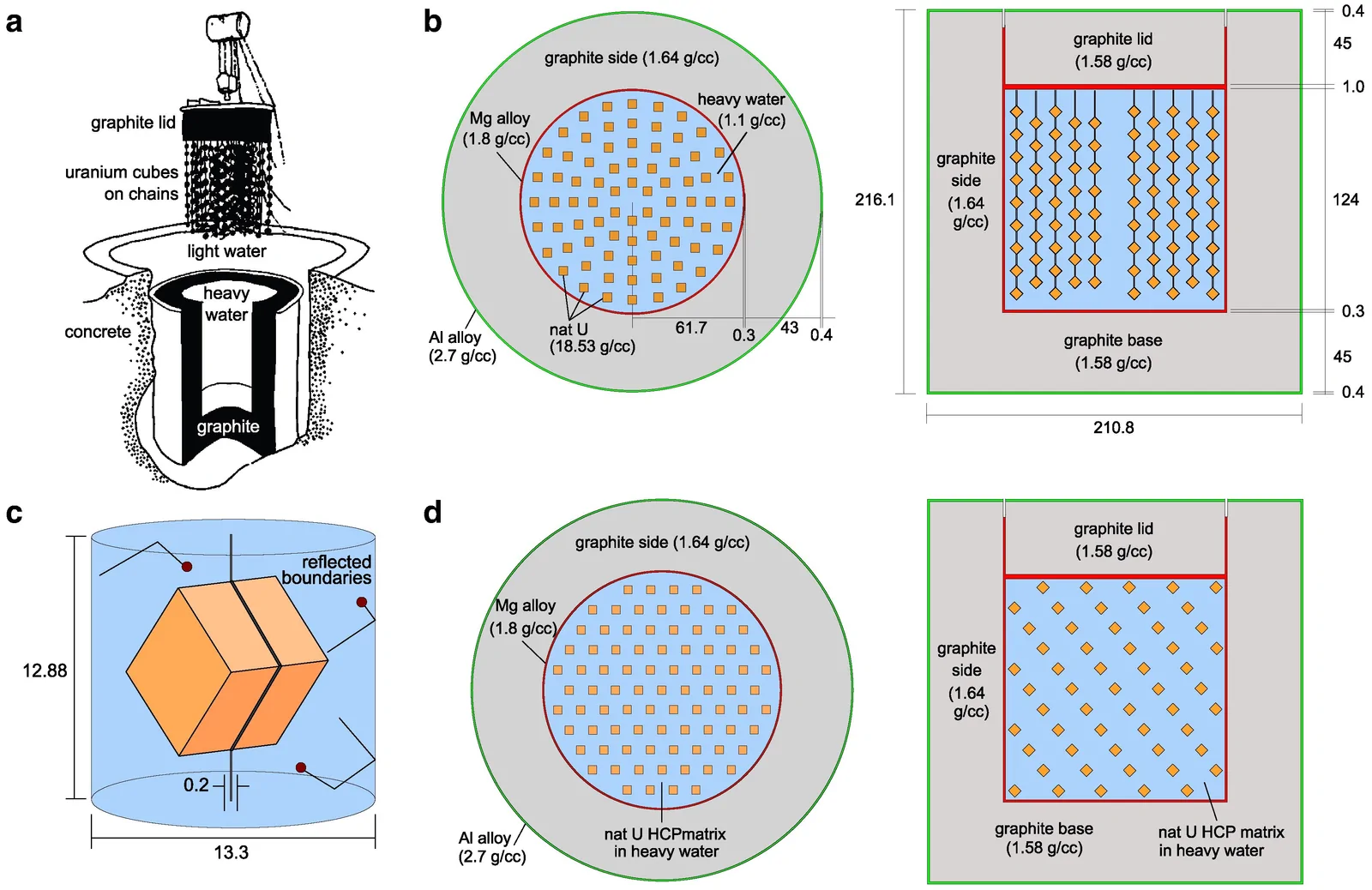
a) Cutaway sketch of the B8 pile as built in April 1945; the chains would be lowered into the heavy water. b) A “fiducial” MCNP neutronics model of the B8 as faithfully to how it was built. c) A “unit cell” model of a single cube and the surrounding heavy water with neutrons reflected at the boundaries to represent an infinite lattice. d) A “lattice model” with the fuel hexagonally close-packed to optimize the fuel spacing (pitch) and provide a way of algorithmically enlarging the lattice when finding the critical core size.

## Neutronics benchmarks and analysis

### Criticality

The criticality of B8 calculated from our fiducial model, i.e. as it was physically built in April 1945, is:


(3) $$k_{\mathrm{eff}}=0.94252\pm 0.00011.$$

The percentages of fissions caused by neutrons in the MCNP-defined thermal, intermediate, and fast energy ranges are: 87.46% (<0.625 eV), 4.45% (0.625 eV–100 keV), and 8.09% (>100 keV). The B8 had averages of 2.46 neutrons produced per fission and 1.17 fission neutrons per neutron absorbed in fuel. A neutron energy histogram indicates that the B8 is strongly thermal with the most-probable energy at 0.0533 eV.

The unit cell model yields $k_{\infty }=1.11594\pm 0.00005$, which retrieves $k_{\infty }=1.11$ originally reported by Heisenberg and Wirtz, validating our model of the fuel and moderator (12). Dividing $k_{\mathrm{eff}}/k_{\infty }$ gives a nonleakage factor L of 0.84, i.e. about 16% of all neutrons are lost outside the U-${\text{D}}_{2}\text{O}$ core, which is exceedingly high and speaks to its small size and graphite quality.

### Heisenberg’s multiplication factor, *Z*

Heisenberg measured Z=6.7 as defined in Eq. 1 but did not provide any error bars. Based on his raw neutron counts ($N_{0}=16.9$, $N_{a}=113.4$), Pešić in Ref. (9) estimated a 1σ uncertainty from Poisson counting statistics of Z=6.7±1.75.

To reproduce *Z* in MCNP, we use an F1 surface current tally on the inside surface of the Mg shell, recording the net number of neutrons leaving the U-${\text{D}}_{2}\text{O}$ core per source neutron. Since essentially no neutrons are produced by graphite, Mg, Al, or water beyond the core—these materials only absorb and scatter—the net thermal current at this boundary is the total source term for the regions Heisenberg integrated over to determine $N_{a}$ and $N_{0}$.

Because the core-empty measurement ($N_{0}$) lacks ${\text{D}}_{2}\text{O}$ moderation, its outgoing neutron spectrum is harder than that of the loaded case ($N_{a}$). To account for this spectral difference and simulate Heisenberg’s detector response, we histogram the energies of outgoing neutrons and weight them by the Dy-164(n,γ) cross section. Using the fiducial model, we obtain:


(4) $$Z_{\mathrm{MCNP}}=6.465\pm 0.003.$$

Without the Dy-164 weighting, the raw tally gives Z=6.112±0.003. That the Dy-weighted value is necessary to match Heisenberg’s result confirms the spectral mismatch matters and that our weighting better accounts for it. Both values are comfortably within the first standard deviation from the counting statistics (±1.75) and give strong confidence to our $k_{\mathrm{eff}}$ calculations against the experimental metric *Z* that the Germans used.

### Nuclear lengths

Average pitch, i.e. center-to-center distance between cubes, is 14.89 cm. If we account for cube thicknesses by subtracting the diameter of a sphere of equivalent volume, 6.20 cm, then the displacement neutrons have to moderate is only roughly 8.69 cm. To examine if this is near the optimal slowing-down length for neutrons in the B8’s heavy water, we simulate a sigma pile experiment in MCNP.

A sigma pile models neutron diffusion and absorption along a column of material. Here, Watt-spectrum fission neutrons are diffused through a column of 96.8 mol% ${\text{D}}_{2}\text{O}$ using MCNP. Neutron current histograms track cumulative captures at various depths. At shallow depths, neutron population slightly rises due to (n,2n) reactions. As neutrons thermalize below 0.625 eV, capture by ${\text{D}}_{2}\text{O}$ and ${\text{H}}_{2}\text{O}$ increases. Thermal neutron fraction peaks at 17.88 cm, nearly double the B8’s fuel spacing, suggesting criticality could improve if the cubes were further distanced.

### Sensitivity analysis

We assess $\Delta k_{\mathrm{eff}}$ from uncertainties in uranium density and heavy water purity through a sensitivity analysis, shown in Fig. 2. Varying uranium density from our measured 18.53 g/cm${\;}^{3}$ to the nominal 19.01 g/cm${\;}^{3}$ changes $k_{\mathrm{eff}}$ by only 0.00037±0.00022 (0.04%). By contrast, increasing heavy water purity from 96.8 to 100 mol% raises $k_{\mathrm{eff}}$ by 0.00774±0.0002 (1.3%). Thus, Fig. 2 provides the $\Delta k_{\mathrm{eff}}$ by which to shift our $k_{\mathrm{eff}}$ and $k_{\infty }$ calculated here should future archaeological evidence revise our specifications.^g^

**Figure 2:**
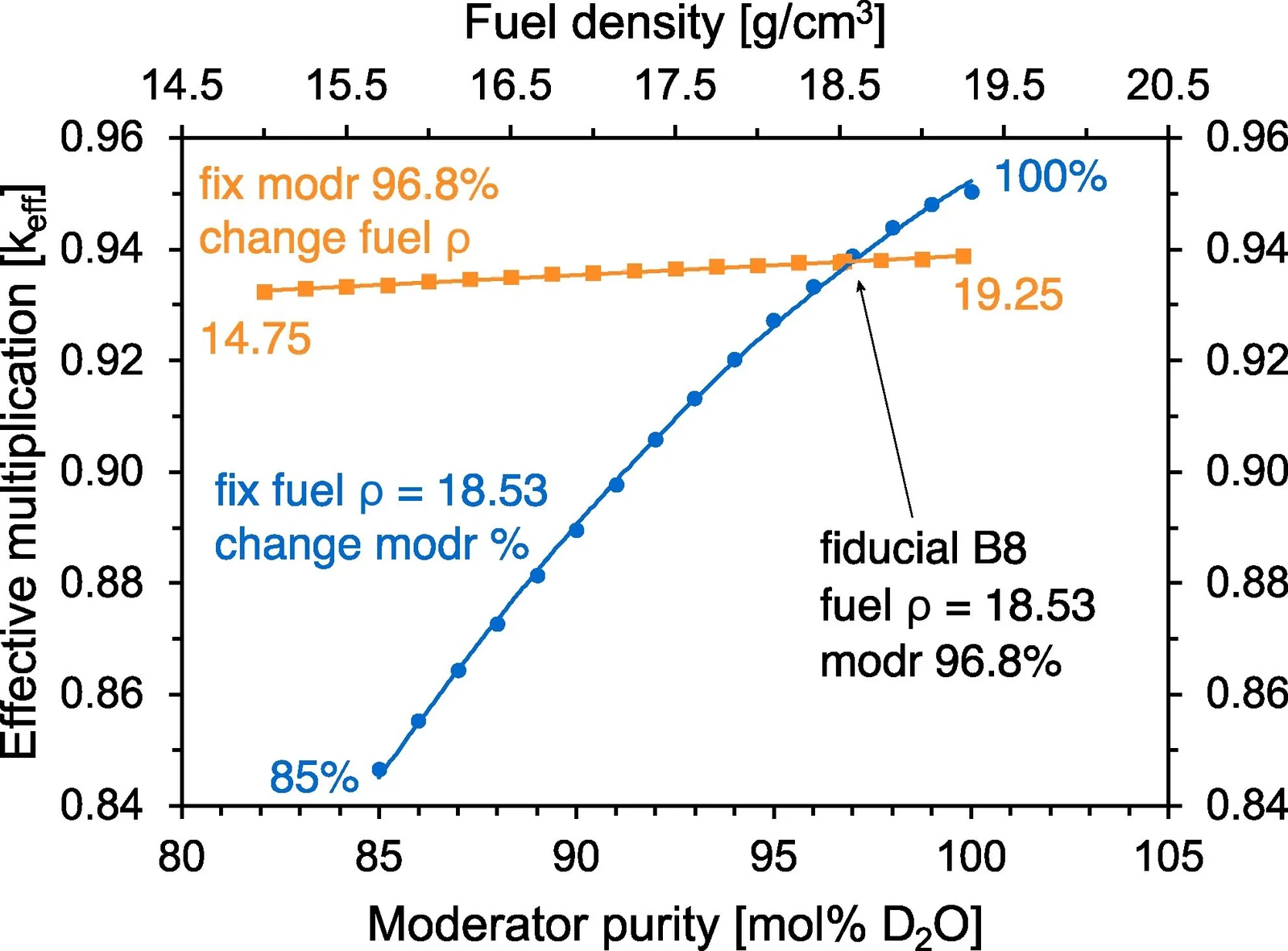
Sensitivity of the effective multiplication factor $k_{\mathrm{eff}}$ to uranium density and moderator purity. First, the NU density at 18.53 g/cm${\;}^{3}$ is fixed and moderator purity is perturbed (circle), and then vice versa (square). Even if 100%-pure D_2_O was used in the B8, $k_{\mathrm{eff}}$ only increases by 0.00774, from the fiducial $k_{\mathrm{eff}}=0.94252$ to 0.95026. (From MCNP with 200k neutrons/cycle; ±3σ errors <1%.)

## Criticality optimization

So far, we have determined that the B8 was a thermal reactor with $k_{\mathrm{eff}}=0.94252$ and $k_{\infty }=1.11594$. We can now calculate the optimal moderator-to-fuel ratio for the B8’s natural uranium-heavy water configuration, and use that information in conjunction with the k_∞_ to predict its critical size.

### Moderator-to-fuel ratio

The suboptimal pitch implies the B8 was undermoderated, i.e. had too low of a moderator-to-fuel volume ratio ($V_{M}/V_{F}$).^h^ The ratio of materials in the fiducial B8 yields a $V_{M}/V_{F}$ of 13.3 and $k_{\infty }=1.11594$. We now find the $V_{M}/V_{F}$ that maximizes $k_{\infty }$ using the unit cell model shown in Fig. 1(c). Keeping the 5-cm uranium cube unchanged, we incrementally increase the volume of the moderator around the cube. Because the unit cell model has reflected boundaries, it simulates having identical cells of fuel above, below, and next to it, such that enlarging the unit cell emulates increasing the lattice pitch. Figure 3 shows our results. The optimal $V_{M}/V_{F}$ for the B8’s natural uranium fuel and 96.8 mol% ${\text{D}}_{2}\text{O}$ moderator is 18.6, yielding the highest possible k_∞_ of 1.1271. This verifies that the B8’s material composition *allows* criticality given a sufficient size of the core.

**Figure 3:**
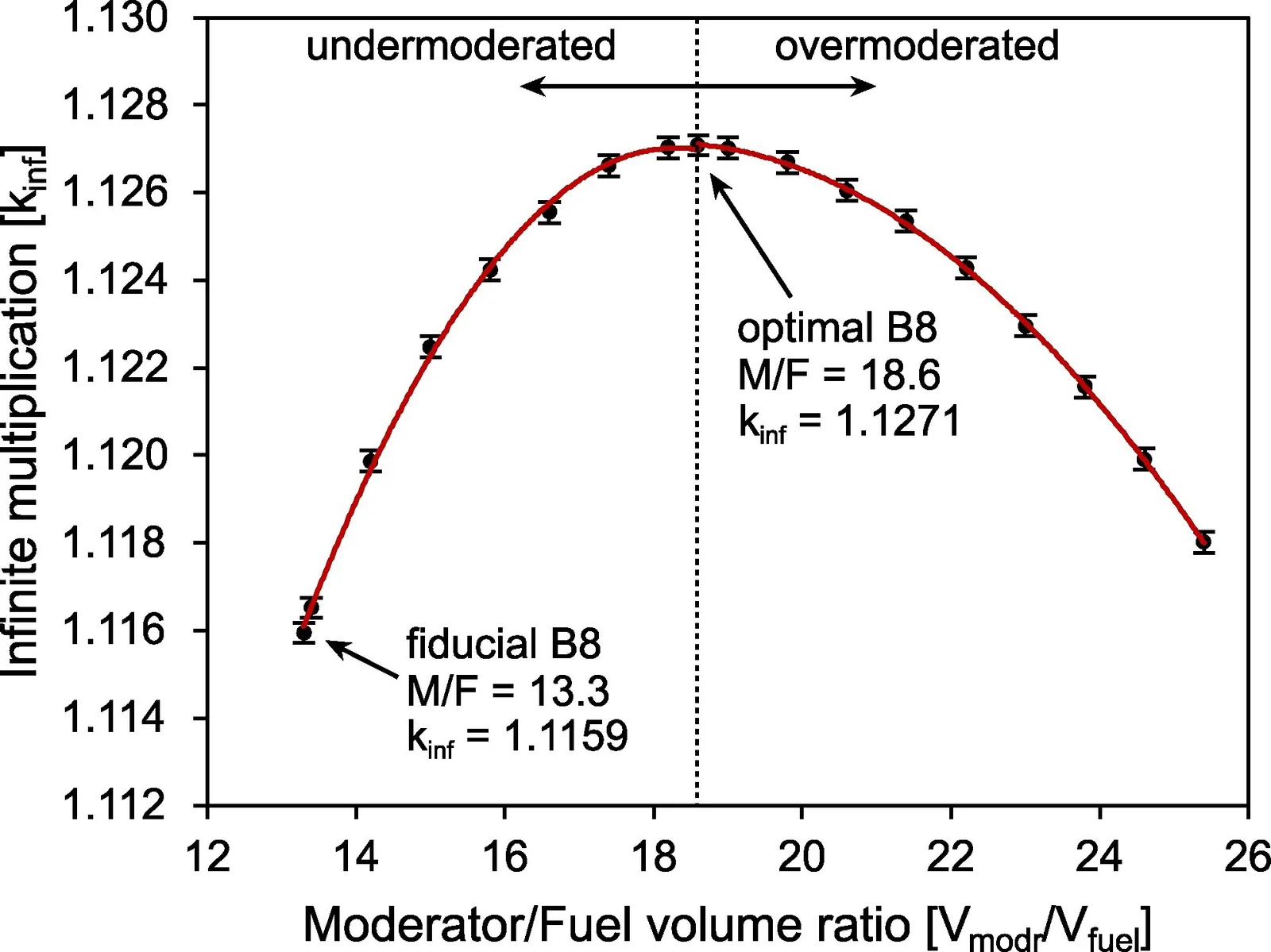
The fiducial B8 had a suboptimal volume ratio of moderator to fuel $V_{M}/V_{F}=13.3$. If the core size was increased, thus increasing the average heavy water moderator volume between uranium cubes, to our calculated optimal $V_{M}/V_{F}=18.6$, then the neutron multiplication factor *k* would increase by 0.0112, from the fiducial $k_{\infty }=1.1159$ to 1.1271. (From MCNP with 200k neutrons/cycle, 100 cycles; error bars ±3σ.)

### Critical size

Now, we solve the nonlinear optimization of minimizing uranium and heavy water inventories for a critical B8. Owing to the nonconvex feasible space, we employed a heuristic search algorithm following the Morris method to trace Pareto-optimal frontiers. To ensure the solution space was systematically explored, for N∈[500,2,100] cubes, we arranged them in a hexagonal close-packed lattice and then scaled them to fill a target moderator volume V∈[1,200,4,000] L. Then this core was surrounded by 40 cm of B7 graphite and then by 60 cm of light water. Such fuel-moderator combinations yielded 1,222 unique MCNP models, each run with 120k neutrons for 100 cycles.

Figure 4 linearly interpolates our 1,222 $k_{\mathrm{eff}}$ results to project criticality over continuous combinations of fuel-moderator inventories. Contour inflections coincide with underfilled edge layers creating local suboptimalities. Although the fiducial B8 was undermoderated, it lay close enough to the optimal $V_{M}/V_{F}$ such that changes in either fuel or moderator inventory mostly lower $k_{\mathrm{eff}}$ without a large change in the other. Adding roughly 100 L more moderator to the fiducial B8 inventory (from 1,400 L to 1,500 L) yields the only improvement, as any more heavy water overmoderates the core. Varying cube counts at a fixed 1,400 L moderator offers no marginal gains, if not decreases, criticality.

**Figure 4:**
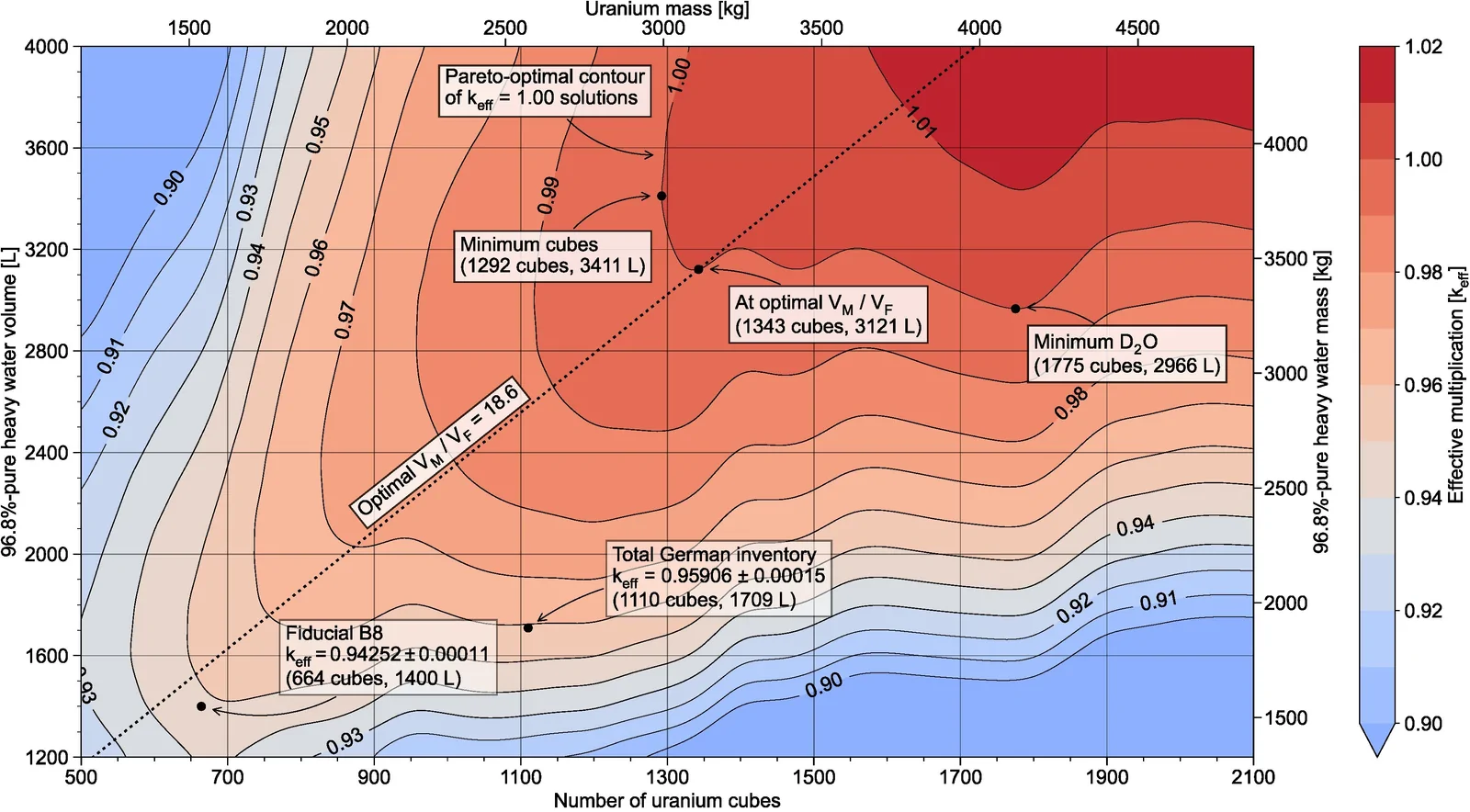
Contour map of the B8’s criticality as functions of the number of natural uranium cubes and liters of 96.8 mol% D_2_O moderator. Even if all the uranium and heavy water available in Germany were combined, the B8 would be subcritical ($k_{\mathrm{eff}}\approx 0.96$) and need hundreds of more cubes and at least a thousand liters more heavy water. Criticality $k_{\mathrm{eff}}=1$ only occurs across a Pareto front with minima at either 1,292 cubes (2,994 kg U) + 3,411 L (3,772 kg) of moderator (minimum-fuel case) or 1,775 cubes (4,113 kg U) + 2,966 L (3,281 kg) of moderator (minimum-moderator case). The dotted spline is the previously calculated optimal moderator-to-fuel volume ratio of 18.6, which intersects the $k_{\mathrm{eff}}=1$ contour at 1,343 cubes (3,111 kg U) and 3,121 L (3,453 kg) of moderator. (Contours interpolated from $k_{\mathrm{eff}}$ calculations of 1,222 unique HCP-lattice MCNP models, each run with 120k neutrons for 100 active cycles; 1σ≈±0.00015.)

Table 1 summarizes the important results. The $k_{\mathrm{eff}}=1$ criticality frontier has a minimum-fuel case at 1,292 cubes (2,994 kg) and 3,411 L (3,772 kg) moderator, as well as a minimum-moderator case at 1,775 cubes (4,113 kg) and 2,966 L (3,281 kg) moderator. The optimal $V_{M}/V_{F}=18.6$ ratio we calculated allows criticality with 1,343 cubes (3,111 kg) and 3,121 L (3,453 kg) moderator. For the German pile groups’ total uranium cube and heavy water inventory of 1,110 cubes and 1,709 L moderator, Fig. 4 projects $k_{\mathrm{eff}}\approx 0.96$. *To go critical, the B8 needed about 2 times the natural uranium and about 2.3 times the 96.8%-pure heavy water than what was actually used in the 1945 experiment.*

**Table 1:** Calculated critical configurations of natural uranium and heavy water (96.8 mol% $D_{2}O$) compared with the B8 as built and the estimated total German wartime inventories.

Configuration	Uranium (kg)	Heavy water (kg)	$k_{\mathrm{eff}}$	NU/B8	${\text{D}}_{2}\text{O}$ /B8
B8 as built	1,538	1,549	0.9425	1.00×	1.00×
Total German inventory	2,572	1,890	0.9591	1.67×	1.22×
Critical: minimum fuel	2,994	3,772	1.000	1.95×	2.44×
Critical: optimal $V_{M}/V_{F}=18.6$	3,111	3,453	1.000	2.02×	2.23×
Critical: minimum moderator	4,113	3,281	1.000	2.67×	2.12×
Chicago Pile 3 (32, 33)	2,493	3,767	1.000	1.67×	2.43×

Uranium masses assume the measured B8 cube density of 18.53 g/cm${\;}^{3}$; moderator masses are for 96.8 mol% ${\text{D}}_{2}\text{O}$. The critical size of Chicago Pile 3 (1944) are also reported for comparison.

## Alternate graphite configuration

Because of the B8’s subcriticality, historians have suggested the Uranverein should have pursued graphite-moderated reactors instead. For instance, P. L. Rose argued for the graphite pathway, saying “heavy water proved awkward on various counts and undoubtedly slowed down the German project” (34).^i^ Even Richard Rhodes opined, “it was crucial that…you needed pure graphite. Evidently, the Germans didn’t understand that for reasons I’ve not seen in the record. It seems to have been a mistake in [developing] the technology.” (4).

With our prior work on Siemens electrographite and our archival findings of the B7’s graphite impurities, we can now calculate whether available German graphites could successfully moderate a natural uranium pile to criticality. In MCNP, we create a cuboid unit cell of a 5 cm uranium cube surrounded by graphite, with reflected boundaries, and measure the $k_{\infty }$ as a function of the cell length. We tested two kinds of uranium: nominal natural uranium (NU, with density 19.01 g/cm${\;}^{3}$ and no impurities) and Degussa NU used in the B8 (18.53 g/cm${\;}^{3}$, 7.1 wppm EBC), with four graphites: nominal pure graphite (1.7 g/cm${\;}^{3}$, no impurities), AGOT graphite (1.6 g/cm${\;}^{3}$, 1.4 wppm EBC), B7 graphite (1.7 g/cm${\;}^{3}$, 3.4 wppm EBC), and 1941 Siemens electrographite (1.7 g/cm${\;}^{3}$, 6.6 wppm EBC). We adopt $k_{\infty }\geq 1.03$ as the practical cutoff for there to exist a realistically sized lattice, of pitch equal to the cell length, that achieves criticality.^j^

Figure 5 shows our results. With Degussa uranium used in the B8 and either 1941 Siemens electrographite or the B7 graphite, no critical pile is possible. Graphite purity is more decisive than that of uranium: reducing graphite boron impurities from 3.4 wppm (B7) to 1.4 wppm (AGOT) raises $k_{\infty }$ by 0.038 at 18 cm pitch, roughly 2–3 times the gain from purifying the uranium alone (B8 vs. pure NU). The historical implication is that the Uranverein’s graphite path was foreclosed by industrial materials bottlenecks. Only purifying uranium metal or access to AGOT-like coke feedstocks, as in the United States, could have made a graphite-moderated pile feasible. The reason for the large criticality difference between Siemens and AGOT graphites is because of the differences in production methods described in our Forensics section and our prior work (26).

**Figure 5:**
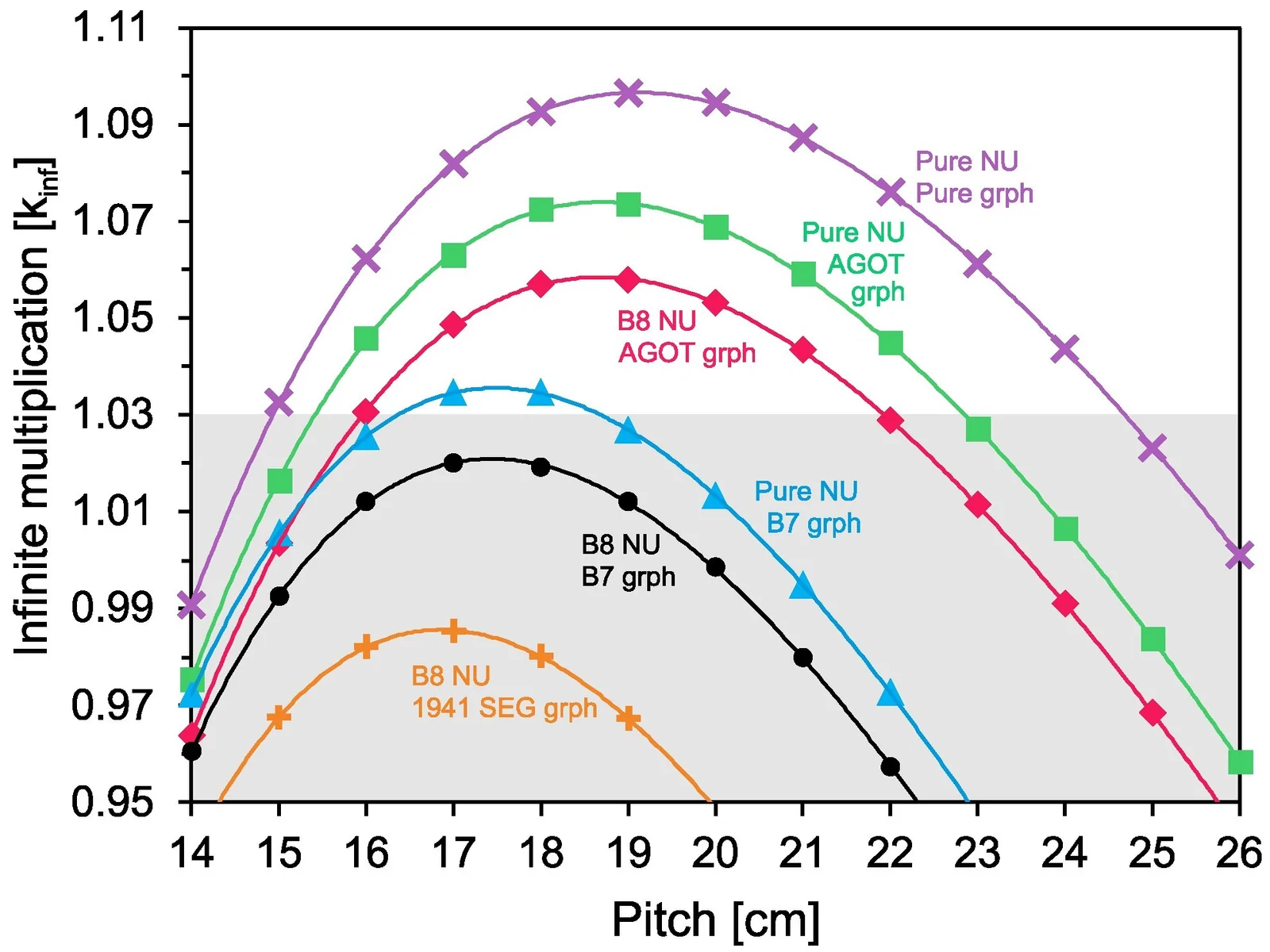
Criticality is predicted for various lattice pitches of counterfactual natural uranium (NU) and graphite (grph) combinations. In this plot, a graphite pile is critical if its $k_{\infty }\geq 1.03$ (see earlier footnote). The lower two curves show a critical pile is impossible with any uranium and graphite the Germans had on hand. The middle two curves show pure uranium or purer graphite are necessary for a critical pile. (B7 = reactor experiment preceding B8; SEG = Siemens electrographite; AGOT = purified graphite made in 1942 by the Manhattan Project. From MCNP with 200k neutrons/cycle, 100 active cycles; ±3σ errors <1%.)

### Comparison to prior works

Grasso et al. and Pešić have done prior criticality calculations of the B8, albeit using various assumptions of materials that we have now clarified here (8, 9). Grasso et al. claimed the B8 had $k_{\mathrm{eff}}=0.85$, based on an assumption that Heisenberg’s Z=6.7 is equivalent to the modern subcritical multiplication factor *M*. This *M* is the ratio of the total neutron population sustained by an external source in a subcritical core to the source alone:


(5) $$M=\frac{1}{1-k_{\mathrm{eff}}},$$

but there is no bijective mapping of *M* to *Z*. *M* is dependent on the effective neutron multiplication eigenmode within the core. *Z* compares the measured thermal neutron population outside the core with and without fuel and moderator. Despite this, Grasso et al. produced $k_{\mathrm{eff}}=0.85$ with a semi-homogeneous model. It is unclear how this was achieved, as homogenization would also underestimate $k_{\mathrm{eff}}$, due to a lower resonance escape probability and lower fast fission rate.

Pešić predicted $k_{\mathrm{eff}}=0.95310\pm 0.00017$ with a full heterogeneous model, assuming nominal 19 g/cm${\;}^{3}$ uranium density and 95%-pure D_2_O (9). Our calculations align nicely, as his slightly higher uranium density and lower D_2_O purity effectively cancel out relative to our forensic specifications. While proposing an error bound to *Z*, Pešić accepted Grasso et al.’s presumption of Z=M and thus did not attempt to reproduce *Z* in the manner described in the primary source.

While Pešić constructed a good heterogeneous MCNP model, his material specifications were necessarily assumed—nominal uranium density, estimated ${\text{D}}_{2}\text{O}$ purity, and graphite impurities selected from a generic range—because the forensic and archival data were not yet available. A central contribution of our work is the nuclear archaeology itself: tracing the uranium cube density to a measured 18.53 g/cm${\;}^{3}$ via helium pycnometry, recovering the only known ${\text{D}}_{2}\text{O}$ purity measurement (96.8 mol%) from an extant NIST dewar, tabulating the total wartime heavy water inventory from Norsk Hydro production records, and benchmarking the B7/B8 graphite impurities (3.4 wppm EBC) from archival ash analyses. They are independently verified inputs that, for the first time, allow the model to reproduce Heisenberg’s in situ measurements (Z=6.7 and $k_{\infty }=1.11$) without tuning free parameters. This reproduction has archaeological value in its own right in-between the historical record and modern simulation, providing confidence that subsequent calculations—our criticality optimization over 1,222 configurations and our graphite pathway analysis, neither of which Pešić nor Grasso et al. attempted—rest on a validated foundation, rather than on assumed inputs.

### Was Germany close to a reactor?

Our optimization furthers the literature by contextualizing how far the fiducial B8’s $k_{\mathrm{eff}}=0.95$ truly is from criticality. Prior historical and technical works tend to describe this *k*_eff_ to be claimed as “not so far” from $k_{\mathrm{eff}}=1$. In addition to the uranium, we demonstrate the B8 would have required about twice the heavy water that was historically produced by Norsk Hydro during the entire war (1,836 kg). The gap between $k_{\mathrm{eff}}=0.94$ and $k_{\mathrm{eff}}=1.0$ may appear small numerically, but the material cost of closing it would have far exceeded Germany’s total production capacity—a distinction that prior accounts have elided.

We further check our prognosis with Chicago Pile 3, a similar heavy water-moderated, natural uranium reactor built by the Manhattan Project in 1944.^k^ For 2.5 metric tons of natural uranium metal rods, CP-3 needed 3.8 tons of heavy water to achieve criticality (32, 33). The B8’s higher fuel demand reflects the relatively high impurity (7.1 wppm EBC) and subnominal density of German cast natural uranium metal.

On the graphite path, our graphite lattice calculations show that Siemens-grade graphite could not attain $k_{\infty }>1.03$ with natural uranium cubes at any plausible pitch. The AGOT graphite used in Chicago Pile 1 combined access to exceptionally low-boron petroleum coke with thermal purification and several handling process controls at Oak Ridge, to achieve nearly pure graphite. The availability of petroleum coke-based graphite was decisive for CP-1 and was not reproducible in Germany after the January 1940 blockade of US petroleum coke (26, 27, 31).

Even if the Uranverein could make an AGOT-like graphite, the outlook for German graphite reactors still would not necessarily be better. The economic, logistical, and physical infrastructure necessary to produce reactor-grade graphite is significant, and at no point did the Uranprojekt ever crystallize into the technoeconomic leviathan the Manhattan Project would grow to be. Some part was, ironically, because the very nature of a nuclear program ran contrary to Germany’s industrial priorities. Even in 1937, Germany faced severe austerity across all sectors under Hitler’s rearmament programs; by 1941, steel output met only half the military’s needs while coal, oil, and coke—with which steel, aluminum, graphite are made—was in deficits of millions of tons. Prioritizing immediate rearmament caused railways to be neglected and deteriorated, causing in the late-war a “transportation calamity” where mountains of mined ores were undelivered, which Göring called “*the* problem of the war economy” (35). In this context, it is debatable if Siemens, whose 15,000 tons per annum graphitization capacity was already demanded for steelmaking, could produce the 5,000 tons of graphite necessary for Hanford-sized plutonium breeder reactors (36). The finished graphite would then require rolling stock to be transported to a safe location for assembly and operation. Several counterfactuals may seem feasible if one only sets total demand against total supply on article, but to practically achieve them in wartime conditions would require nearly omniscient foresight and coordination.

## Conclusions

Werner Heisenberg estimated in 1947 that “a relatively small amount of uranium” would suffice for the B8 pile to achieve criticality (3), and refined this in 1948 to increasing the core volume “by not quite half”—corresponding to roughly 1.5 times the uranium and heavy water—would have been required for the B8 pile to achieve a self-sustaining neutron chain reaction, i.e. $k_{\mathrm{eff}}=1$. Our calculations suggest Heisenberg significantly underestimated the materials required. We calculate the original B8 achieved around $k_{\mathrm{eff}}=0.94252$. At minimum, the B8 would have required either 1,292 cubes with 3,411 L of heavy water (minimal fuel) or 1,775 cubes with 2,966 L of heavy water (minimal moderator). These represent an increase of 1.9 to 2.7 times as much uranium and 2.1 to 2.4 times more heavy water volume than was used, far exceeding Heisenberg’s claims. We also find that no natural uranium, graphite-moderated pile could have gone critical with the uranium and Siemens graphite available to the Uranverein.

We hope our benchmark of the critical dimensions of the B8, as well as a hypothetical Siemens graphite pile, prove useful in future historical assessments of the German nuclear program. By revisiting historical pile designs with precision, we can better understand the technical challenges faced by early, even failed, nuclear programs and more accurately assess their capabilities and limitations. Our work could be a template to analyze other historical reactors, creating a comparative framework for understanding the technical evolution of reactor design across different national programs.

## Methods

### Cubes

A total of 14 of the uranium cubes used in the nuclear reactor experiments in Germany are known to exist today. They are held in private and museum collections in the United States and Europe. Most of these surviving cubes, including those analyzed in this work, can be traced directly back to the individuals who removed them from Germany during the war, providing proof of their provenance. Based on the limited available records, it is believed that the majority of the uranium cubes used in B8 were transported to the United States where they were melted down and used in the production of the country’s nuclear stockpile (37).

### Monte Carlo models

Monte Carlo N-Particle (MCNP) code is an industry-standard, general-purpose Monte Carlo particle transport code for nuclear reactor analysis. We used continuous-energy cross sections from Evaluated Nuclear Data Files, version B-VIII.0, with boron corrections, temperature interpolation of cross sections via pseudomaterials in the data cards, and S(α,β) kernels at 293 K (38).

The dimensions of the unit cell model are computed from the Voronoi decompositions of the fuel lattice in the fiducial model. Using the Python scipy package, the average Voronoi volume was 1,787.75 cm${\;}^{3}$ and surface area in the XY plane (plan view) 138.82 cm${\;}^{2}$. Thus the unit cell has radius 6.65 cm and height 12.88 cm.

For the HCP lattice model, since it is a regular 3D lattice, we generated lattice positions by mapping integer coordinates $\vec{n}=(i,j,k{)}^{⊤}$ on a simple cubic lattice to physical HCP positions $\vec{r}=(x,y,z{)}^{⊤}$ (in units of the lattice parameter *a*) via $\vec{r}=A\vec{n}$, where A is the basis matrix:


(6) $$\left[\begin{array}{c} x\\ y\\ z \end{array}\right]=\left[\begin{array}{ccc} 1 & 1/2 & 1/2\\ 0 & \sqrt{3}/2 & \sqrt{3}/6\\ 0 & 0 & \sqrt{2/3} \end{array}\right]\left[\begin{array}{c} i\\ j\\ k \end{array}\right].$$

The columns of A are the three primitive vectors of the HCP lattice; the first two generate the triangular layers and the third staggers successive layers. We sorted the resulting cube centers by the L2 norm along the radius and height. In order to construct an *N*-cube cylindrical lattice model, we took the first *N* points from the sorted list and scaled the coordinates such that each cube center is at least $5\sqrt{3}/2\approx 4.5$ cm away from the reactor boundaries.

We choose to arrange the fuel in a lattice when modifying the B8’s fuel-moderator inventories for geometric convenience. Enlarging the fiducial B8 requires multidimensional decisions to arrange *N* arbitrary cubes, such as the vertical pitch along the chain, the number of chains per ring, and the pitch between rings. In contrast, the HCP lattice model collapses the decision space in arranging *N* cubes to a single variable—the lattice parameter *a*—as HCP has a fixed height-to-edge ratio $c/a=\sqrt{8/3}$. For a fixed fuel fraction and core size, prior work has determined that fuel in HCP and FCC lattices, which are equally the highest-density solutions to the sphere packing problem, also produce the same $k_{\mathrm{eff}}$ within typical Monte Carlo uncertainties of 0.1–0.2% (39). We ensure our HCP core model reproduces the criticality of the fiducial core: the HCP model for 664 cubes and 1,400 L moderator has $k_{\mathrm{eff}}=0.94307$, only a 0.027% difference from the fiducial B8’s 0.94252 in Eq. 3. Thus, an HCP lattice was a computationally reasonable way to parametrize modifying the B8 geometry.

## Data Availability

All neutronics code and data to support our claims are freely available at: github.com/patrickpark910/b8pile.

## Notes

a Heisenberg wrote the B8 used about “1.5 tons” of heavy water, which some secondary histories quote as 1,500 L, but D_2_O has density 1.1 g/cm${\;}^{3}$, so the volume is more like 1,400 L.

b The full expression in Ref. (10) include various correction factors, e.g. *Y* for fast fission and exp(−w) for resonance capture during thermalization, omitted here for brevity.

c Physicists of the era often used cadmium or dysprosium absorption measurements to experimentally define “thermal” neutrons, not realizing those metals had resonances above 1 eV. This caused systematic underestimation of thermal capture measurements in other materials, including the pre-war cross-section data for carbon and boron (26). Heisenberg’s ${\text{Dy}}_{2}{\text{O}}_{3}$ probes introduce a similar concern. His *N*’s meant to indicate thermal neutron populations. However, Dy isotopes have intermediate-energy resonances, so the probe would count more epithermal neutrons than a pure 1/v absorber. This creates a bias when comparing $N_{a}$ and $N_{0}$ measurements, as the empty-core neutron spectrum is harder than when neutrons are moderated by ${\text{D}}_{2}\text{O}$. A second error occurs when using $\nu_{C}$ to convert graphite measurements to light water-equivalents. We are unsure if the Germans were aware of Dy resonances; Manhattan physicists only discovered this error in 1942 (40).

d Heisenberg does not give the precise positions of his ${\text{Dy}}_{2}{\text{O}}_{3}$ detectors. He reported calculating their positions by “usual Gaussian quadrature” (presumably Gauss-Legendre mapped to cylindrical coordinates), but this yields detector positions implausibly close to the heavy water-graphite interface. Thus, Ref. (13) should be read carefully before attempting to reproduce *Z*.

e These dewars remain at the National Institute of Standards and Technology (37).

f Germany also had a Bavarian natural graphite mine in the 1940s that is still in operation today by Graphit Kropfmühl. Its boron content was not measured, but judging from its average iron content, it is most unlikely that it could moderate a reactor without modern purification methods (41).

g Formally, reactivity $\rho =(k_{\mathrm{eff}}-1)/k_{\mathrm{eff}}$ is additive, but ρ≈Δk in our range of *k* values.

h A higher $V_{M}/V_{F}$ can improve neutron thermalization and reduces migration length, but excessive moderation leads to increased thermal absorption in the moderator.

i Rose also asserts that by 1944, Fünfer and Bothe calculated the “true” capture in carbon since graphite was used in the B6 pile. This likely misunderstands Refs. (3, 31, 42). The B6 only confirmed Siemens electrographite as a suitable reflector. A reactor’s reflector, which reduces neutron leakage, is less sensitive than its moderator, which directly affects chain reaction efficiency.

j Although criticality in principle requires only $k_{\infty }>1$, even a large reactors like CANDU realistically requires $k_{\infty }\geq 1.03$ to overcome penalties of a few percent from neutron leakage and structural absorption such that its *effective* neutron multiplication ratio $k_{\mathrm{eff}}$ reaches 1.0 (43).

k Without an equal to Norsk Hydro, the Americans built their own plants via the P-9 Project to produce hundreds of tons of D_2_O by the end of the war (44). An ANL report (32) gives CP-3’s nominal natural uranium and heavy water loading, while the Smyth Report (33) tells us it achieved criticality with “only three-fifths of the heavy water,” giving us the critical size used here.
